# Viral Subversion of the Nuclear Pore Complex

**DOI:** 10.3390/v5082019

**Published:** 2013-08-15

**Authors:** Valerie Le Sage, Andrew J. Mouland

**Affiliations:** 1 Lady Davis Institute, Jewish General Hospital, McGill University, Montreal, Quebec H3T 1E2, Canada; 2 Department of Medicine, McGill University, Montreal, Quebec H3T 1E2, Canada

**Keywords:** viruses, nuclear pore complex, nucleoporins, nucleocytoplasmic transport

## Abstract

The nuclear pore complex (NPC) acts as a selective barrier between the nucleus and the cytoplasm and is responsible for mediating communication by regulating the transport of RNA and proteins. Numerous viral pathogens have evolved different mechanisms to hijack the NPC in order to regulate trafficking of viral proteins, genomes and even capsids into and out of the nucleus thus promoting virus replication. The present review examines the different strategies and the specific nucleoporins utilized during viral infections as a means of promoting their life cycle and inhibiting host viral defenses.

## 1. Introduction

The nuclear envelope (NE) acts as a formidable barrier that compartmentalizes eukaryotic cell genomes in the nucleus and enables the uncoupling of transcription and translation. The NE is studded with nuclear pore complexes (NPC), multiprotein channels that regulate selective nucleocytoplasmic trafficking of macromolecules. In non-dividing cells, the NPC promotes communication between the cytoplasm and nucleus by allowing molecules smaller than 5 nm to passively diffuse through the pore, while macromolecules greater than 40 kDa require active transport [1,2,3].

**Figure 1:**
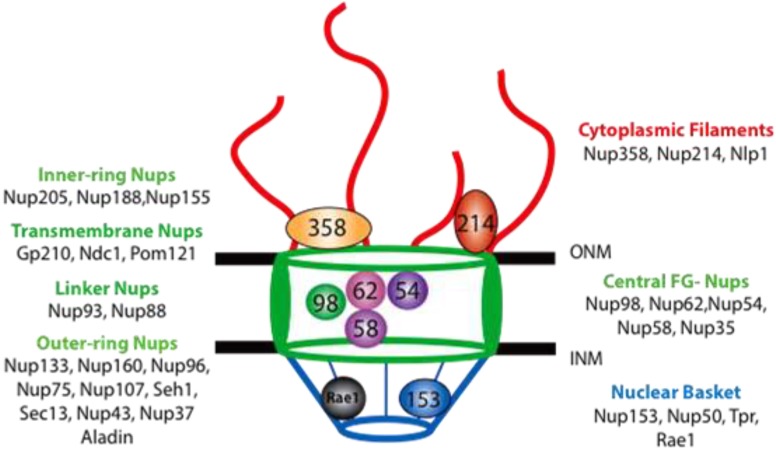
The nuclear pore complex (NPC). Schematic representation of a cross-section view of a NPC as it spans the inner (INM) and outer (ONM) nuclear membranes. Vertebrate Nups are listed based on their localization within the NPC. The central core (green) is composed of the inner-ring Nups, transmembrane Nups, linker Nups, outer-ring Nups and the phenylalanine-glycine (FG)-Nups. The location of select nucleoporins within the NPC is shown.

The NPC is composed of 30 different proteins, referred to as nucleoporins (Nups) [4]. The overall architecture of the NPC and the positioning of each Nup has been determined through molecular, biochemical and structural analysis of the *Saccharomyces cerevisiae* NPC [5,6,7]. The NPC is a modular structure that can be divided into three large regions: the nuclear basket, the central core and the cytoplasmic filaments (Figure 1).

The exchange of material into and out of the nucleus is made highly selective by soluble transport receptors called karyopherins (importins or exportins). The directionality of each transport event requires energy and is regulated by the small GTPase Ran, as it cycles between its GTP- and GDP‑bound forms [8]. Nuclear import is driven by α and β subtypes of the importin superfamily, which specifically binds to cargo proteins encoding a nuclear localization signal (NLS) and together this importin-cargo complex translocates through the NPC [9]. In the nucleus, Ran/GTP binds to the importin-cargo complex and causes its disassembly, resulting in the release of the cargo into the nucleoplasm. Conversely, nuclear export requires the formation of a trimeric complex consisting of an exportin, a nuclear export signal (NES)-containing cargo and Ran/GTP. Once transported across the NPC, the trimeric complex dissociates upon interconversion of Ran/GTP to Ran/GDP to deliver the cargo into the cytoplasm.

Viruses are obligate intracellular parasites that manipulate and exploit host cell machineries during infection. Many nuclear transport events involving viral components occur throughout the course of virus replication. Some viruses are solely able to penetrate the nucleus upon NE disassembly during cellular mitosis, while others actively enter the nucleus by subverting the NPC and promoting nuclear import [10,11,12]. As import through the NPC is size restricted, infecting virus particles have evolved different strategies to breach the nucleus. In this review, we describe the different mechanisms of nuclear transport for adenovirus, herpesvirus, picornavirus, orthomyxovirus, rhabdovirus, retrovirus and hepadnavirus and discuss the host cell factors subverted by these viruses during import and export events (Table 1).

**Table 1:** Host factors subverted by different viruses to facilitate transport across the NPC.

Virus	Host factor	Function(s)	Detection method(s)	Reference(s)
Adenovirus	CRM1	Import/Export	Functional analysis with LMB	[13,14]
	Nup214	Import	IF and biochemical interaction	[15,16]
	Nup358	Import	IF	[15]
	Nup62	Import	IF	[15]
	Histone H1	Import	Biochemical interaction and MALDI MS	[16]
	Transportin 1	Import	Biochemical interaction	[17]
	Hsp70	Import	Import block with anti-hsp70 antibodies	[13]
	Importin α	Import	Biochemical interaction	[17]
	NFX1	Export	IF and RNAi	[18]
HSV-1	Importin β	Import	Capsid binding block with anti-importin β antibodies	[19]
	Nup214	Import	Co-IP and shRNA	[20]
	Nup358	Import	Capsid binding block with anti-Nup358 antibodies and siRNA	[21]
	NFX1	Export	siRNA	[22]
	Nup62	Export	Co-IP and pull-down assay	[23]
	Nup153	Import	Microarray analysis	[24]
EBV	Nup62	Unknown	Co-IP	[25]
	Nup153	Unknown	Co-IP	[25]
Poliovirus/ Rhinovirus	Nup62	Blocks host import	Immunoblot analysis of proteolytic cleavage	[26,27]
	Nup98	Blocks host import	Immunoblot analysis of proteolytic cleavage	[28]
	Nup153	Blocks host import	Immunoblot analysis of proteolytic cleavage	[26,27]
Cardiovirus	Nup62	Blocks host import/export	Immunoblot analysis of phosphorylation	[29]
	Nup98	Blocks host import/export	Immunoblot analysis of phosphorylation	[30]
	Nup153	Blocks host import/export	Immunoblot analysis of phosphorylation	[29]
	Nup358	Blocks host import/export	Immunoblot analysis of phosphorylation	[29]
Influenza A	Importin α	Import	*In vitro* binding assays, Co-IP and cocrystal structure	[31,32,33]
	KPNB1	Unknown	RNAi screens	[34,35]
	CRM1	Export	Functional analysis with LMB	[36,37]
	NFX1	Export	RNAi screens and functional analysis using siRNA	[34,38,39,40]
	Nup98	Export	RNAi screens	[39,40,41]
	Nup153	Unknown	RNAi screens	[35,40]
	Nup62	Export	Functional analysis using siRNA and IF	[38]
	p15	Export	*In vitro* binding assays	[39]
	Rae1	Export	*In vitro* binding assays	[39]
	E1B-AP5	Export	*In vitro* binding assays	[39]
VSV	Nup98	Blocks host export	*In vitro* binding assay and MALDI-TOF	[42]
	Rae1	Blocks host export	*In vitro* binding assay and immunoblot	[43]
HIV-1	Nup98	Proviral integration	RNAi and functional studies using shRNA	[44,45,46]
	Nup85	Import	RNAi and functional studies using siRNA	[47,48]
	Nup133	Import	RNAi	[47]
	Nup107	Import	RNAi	[47]
	Nup160	Import	RNAi and functional studies using siRNA	[47,48]
	Nup153	Import and proviral integration	RNAi and functional studies using siRNA	[44,45,46,47,48]
	Nup214	Export	RNAi and functional studies using shRNA	[44,46]
	Nup358	Import	RNAi and functional studies using shRNA	[44,46,47,48]
	Nup155	Import	RNAi and functional studies using siRNA	[47,48,49]
	CRM1	Export	RNAi and *in vitro* binding assay	[49,50]
	TNPO3	Import	RNAi and functional studies using shRNA and siRNA	[46,47,51]
	Nup50	Unknown	Microarray	[52]
	Nup62	Import/Export	Microarray and functional studies using siRNA	[52,53,54]
HBV	Nup153	Import	Co-IP and immunoblot	[55]

## 2. Adenoviruses

Members of the family *Adenoviridae* are non-enveloped, double-stranded DNA viruses. Currently, there are 57 human adenovirus types, which can be categorized into 6 subgroups and cause a range of respiratory, gastrointestinal, urogenital and ocular diseases. The stepwise process of adenovirus genome transport into the nucleus begins with attachment of the virus fiber protein to the host cell coxsackievirus adenovirus receptor (CAR) [56]. Virus internalization by receptor-mediated endocytosis results in virion escape into the cytoplasm and begins the progressive uncoating of the capsid leaving a somewhat intact layer of hexon and penton coat proteins [57]. The remaining coat proteins allow the virion to travel along microtubules to the nucleus by binding, through an unknown mechanism, to the molecular motor, dynein [58]. Stepwise uncoating of the virion may play a role in evading host innate immune responses by limiting exposure of the viral DNA genome before nuclear import is complete [59].

**Figure 2:**
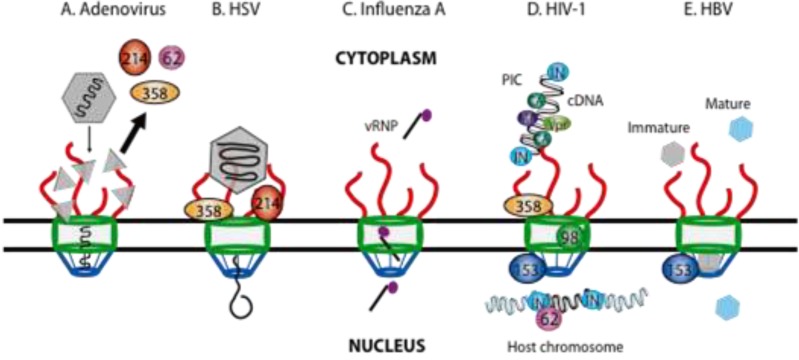
Viruses that hijack the nuclear pore complex to promote viral genome nuclear import. Schematic representations of the different mechanisms by which 5 viruses subvert various Nups in order to facilitate nuclear import. (**A**) Uncoating of the adenovirus capsid at the NPC causes at once the release of the DNA genome into the nucleus and displacement of Nup62, Nup214 and Nup358 into the cytoplasm. (**B**) HSV-1 capsid docking to cytoplasmic filaments leads to uncoating and driving the DNA genome through the NPC. (**C**) Import of the influenza A virus vRNP is mediated by the classical importin α/β transport pathway. (**D**) The HIV-1 PIC (pre-integration complex) docks to Nup358 before being imported into the nucleus and integrated into the host chromosome in the process that is facilitated by Nup62, Nup98 and Nup153. (**E**) Mature and immature HBV capsids are imported intact through the NPC, however; Nup153 ensures that only mature capsids disintegrate to release the DNA genome into the nucleus.

Nuclear targeting of the virus particle requires the presence of the export receptor CRM1 (chromosome region of maintenance 1), as disassociation of the adenovirus virion from microtubules is blocked by the presence of Leptomycin B (LMB), which specifically inhibits CRM1 [14,60]. The final uncoating step occurs as the capsid docks to Nup214 [16] but before disassociating from the kinesin light chain motor [15]. Strunze *et al.* propose a model whereby the kinesin heavy chain is activated upon binding Nup358 (also known as RanBP2) and exerts a pulling force on the viral particle, which disassembles it completely. Indirect immunofluorescence at 3 hours post-infection demonstrates that Nup358, Nup214 and Nup62 are displaced into the cytoplasm and colocalize with disassembled virus particles at the cell periphery (Figure 2A) [15]. The displacement of Nups increases the NE permeability, as evidenced by the transient infiltration of inert fluorescent dextran molecules in adenovirus-infected cells, which presumably promotes entry of the viral DNA into the nucleus [15]. The adenovirus genome is highly negatively charged and consequently a number of viral and host proteins have been implicated in viral DNA import, these include: the viral core protein VII, histone H1, the import receptor transportin 1, hsp70 and importin α [13,16,17,61]. Transport receptors are limiting factors during adenovirus infection and therefore increasing NPC permeability may make viral DNA import less dependent on them [15].

The late phase of the adenovirus life cycle includes viral genome replication, transcription and synthesis of gene products. An increase in the export of viral mRNA transcripts causes a concomitant block in host mRNA export by an unknown mechanism. Interestingly, adenoviral transcripts switch from being exported by CRM1 early during infection to the mRNA export receptor NXF1 during the later phase of viral mRNA export [18,62]. Viral structural proteins are imported into the nucleus for capsid assembly and DNA-filled capsids are released by virus induced cell lysis. Altering the composition of the NPC might not only be involved in viral DNA import but may also be playing an unexplored role in adenovirus modulation of the host in an effort to augment viral replication.

## 3. Herpesviruses

The family *Herpesviridae* is a large group of double-stranded DNA viruses that cause a wide range of disease in a broad range of hosts. The alphaherpesvirus subfamily includes the human pathogens herpes simplex virus (HSV) type 1 and 2, which are responsible for cold sores and genital herpes, respectively. All herpes virions share a common structure; the DNA genome is encased within an icosahedral nucleocapsid that is surrounded by a proteinaceous layer called the tegument and finally surrounded by a lipid envelope embedded with glycoproteins [63].

HSV-1 infection begins with viral glycoprotein (gC or gB) binding to its cognate cellular receptor (heparan sulfate) [64]. Fusion of the virion envelope with the host cell membrane releases the nucleocapsid and the outer tegument proteins into the cytoplasm, whereby the inner tegument proteins interact with molecular motors to transport capsids along microtubules to the nucleus [65]. The prohibitive size of the nucleocapsid taken together with electron microscopy images of accumulating empty nucleocapsids at the cytoplasmic face of the NPC, suggest that incoming herpesviruses have an active mechanism to import DNA into the nucleus (Figure 2B) [66].

This section focuses on HSV-1, which is widely studied in terms of nuclear import. *In vitro* reconstitution of purified capsids and the NPC demonstrate that docking solely necessitates importin β but DNA release into the nucleoplasm additionally requires the presence of cytosol and energy [19]. To date two viral tegument proteins, pUL36 and pUL25, are known to play a role in capsid docking to the NPC. Blocking pUL36 with specific antibodies decreases capsid binding to the NPC [21], and mutation of the pUL36 NLS produces a replication incompetent virus that accumulates capsids at the microtubule organizing center (MTOC) [67]. Biochemical evidence suggests that initial contact between the capsid and the NPC occurs between pUL25 and the phenylalanine-glycine (FG)-repeat domain of Nup214 yet depletion of Nup214 only delays viral DNA nuclear import [20]. Similarly, Nup358 silencing also attenuates capsid attachment to the NPC [21]. Nup358 and Nup214 are known to interact, making it tempting to speculate that they constitute a complex that is targeted by HSV capsids [20]. Once bound to the NPC, pUL36 and pUL25 play a central role in uncoating and DNA release [68,69]. A hypothetical model of DNA translocation suggests that the accumulated pressure within the capsid after initial packaging of the viral DNA is the driving force that propels the genome through the central channel, and as it enters the nucleoplasm, RNA polymerase acts to pull the remaining genome through as transcription occurs [66].

HSV gene expression is temporally regulated and requires the viral protein ICP27 to transport intronless viral mRNA into the cytoplasm. The mechanism of viral mRNA export is dependent upon NXF1 [22] but ICP27 is also able to shuttle independent of NXF1 [23]. Co-immunoprecipitation and *in vitro* binding assays indicate that ICP27 interacts with Nup62 in the absence of viral RNA and other viral proteins suggesting that ICP27 may utilize the NPC directly for its own export [23]. Expression of ICP27 disrupts host protein import by imposing a blockade on both the classical NLS-mediated and the transportin 1/M9 NLS-mediated import pathways through an unknown mechanism [23].

**Figure 3:**
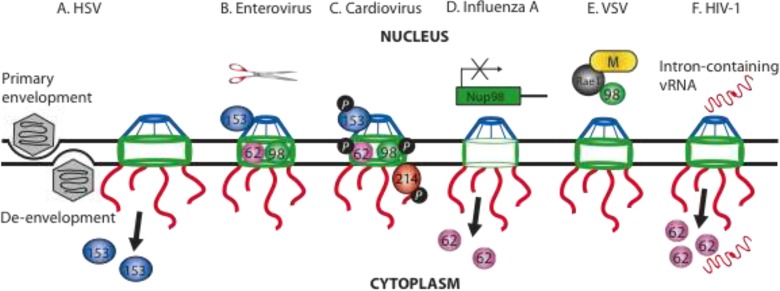
Changes to the NPC by viruses during nuclear export. Schematic representations of the different mechanisms by which 6 viruses subvert various Nups in order to facilitate nuclear export. (**A**) HSV-1 capsids exit the nucleus via a process of primary envelopment and de-envelopment. Secondary effects include dilation of the NPC channel and redistribution of Nup153. (**B**) Enteroviruses replicate in the cytoplasm and induce degradation of Nup62, Nup98 and Nup153 by the protease 2A^pro^ (scissors). (**C**) Cardiovirus infection induces hyperphosphorylation (*P*) of Nup62, Nup98, Nup153 and Nup214. (**D**) Influenza A downregulates the expression of Nup98 and relocalizes Nup62 to the cytoplasm. (**E**) VSV M protein inhibits mRNA export through an interaction with the Rae1-Nup98 complex in the nucleoplasm. (**F**) HIV-1 Rev-mediated vRNA export is CRM1-dependent and causes the displacement of Nup62 into the cytoplasm.

Microarray analysis of mock and HSV-1-infected primary rat embryonic fibroblast reveals that HSV-1 decreases Nup153 gene expression by greater than 3-fold [24], while immunofluorescence of HSV-1-infected cells indicates that Nup153 is dislocated to the cytoplasm at early times post-infection (Figure 3A) [70]. The HSV-1 specific effects on Nup153 may contribute to the blockade in nuclear import, as Nup153 is critical for this process [71]. It has also been suggested that nuclear import is inhibited directly by ICP27 competing with cargo proteins (GFP-M9 and GFP-NLS) for binding to transport receptors [23]. A recent study of the protein kinase BGLF4 from the gammaherpesvirus Epstein-Barr virus (EBV) indicates that BGLF4 binds to Nup62 and Nup153 and appears to induce reorganization of NPC, although functional effects on nucleocytoplasmic transport have yet to be determined [25].

Nucleocapsid assembly and viral DNA packaging occur in the nucleus, making the host transport machinery necessary for import of viral regulatory, capsid and tegument proteins. DNA-containing HSV capsids egress from the nucleus by budding into the inner nuclear membrane thereby acquiring a primary envelope in the perinuclear space (Figure 3A) [72]. The “naked” virion is then released into the cytoplasm through a second fusion event between its primary envelope and the outer nuclear membrane, in a process called de-envelopment (Figure 3A) [72]. Though this NE budding model is now widely accepted, HSV-1 infection appears to have a significant impact on NPC size and architecture (Figure 3A) [70,73]. The HSV assault on the NE, causes NPC channels to dilate to greater than 140 nm without any loss of nuclear material from the nucleoplasm (as seen by high-resolution microscopy [70]) or alterations in total Nups (as visualized by Western blot analysis [73]).

In summary, HSV first hijacks the NPC for genomic DNA import and at early times post-infection blocks host protein import possibly through alteration in the composition of the NPC. At later times post-infection, although HSV egress occurs through the NE the virus appears to cause secondary effects on the NPC channel size, which may have yet to be determined functions in HSV replication.

## 4. Picornaviruses

The picornavirus genome consists of a single-stranded, positive-sense RNA molecule, which is enclosed within an icosahedral capsid. All members of the *Picornaviradae* family encode a single polyprotein that is co- and post-translationally processed by the virus-encoded protease, 3C^pro^. Enteroviruses, such as rhinoviruses and polioviruses (etiological agents of the common cold and polio, respectively) also encode a second protease 2A^pro^, while cardioviruses encode a leader (L) protein. These proteases are not only necessary to generate active viral proteins but are also responsible for modulating a large number of cellular processes through the cleavage of a wide variety of host proteins [74].

The picornavirus life cycle occurs entirely in the cytoplasm and consequently requires the relocalization of a number of host nuclear proteins to promote successful virus replication. In poliovirus-infected cells, many nuclear resident proteins, including La, Sam68, nucleolin and polypyrimidine tract binding protein (PTB) display an aberrant cytoplasmic localization [75,76,77,78]. Poliovirus affects both the classical NLS and transportin 1 import pathways. A green fluorescent protein (GFP) fusion to either the classical NLS (GFP-NLS) or the M9 (GFP-M9NLS) signal sequence localize GFP to the nucleus in uninfected cells, whereas poliovirus-infected cells cause an accumulation of the GFP-NLS and the GFP-M9NLS proteins in the cytoplasm indicating that both protein import pathways are disrupted [26]. However, evidence suggests that CRM1-dependent export remains functional [26] and that mRNAs are exported normally [28]. By contrast, the cardiovirus encephalomyocarditis virus (EMCV) accumulates nuclear proteins in the cytoplasm by increasing the permeability of the NE and by changing existing protein export rather than blocking newly synthesized protein import [79].

Enterovirus and cardiovirus infections alter the composition of the NPC to relocalize a subset of nuclear proteins to the cytoplasm. In poliovirus-infected cells, cargo-receptor complexes are unable to dock at the NPC, which results in the cytoplasmic accumulation of nuclear proteins [26]. The virus‑induced mechanism that forces the selective retention of nuclear proteins in the cytoplasm is associated with alteration in the NPC composition and permeability of the NE. Fluorescence and electron microscopy indicate that the NPC in poliovirus-infected cells contains decreased quantities of Nups [26,80]. The compositional alterations in the NPC are attributed to enterovirus-induced degradation of Nup62, Nup98 and Nup153, which are all critical for nucleocytoplasmic shuttling (Figure 3B) [26,27,28]. Proteolysis of these three FG-Nups have different kinetics with cleavage of Nup98 early, followed by Nup62 and Nup153 at later times post-infection, the latter coinciding with a block in import of multiple nuclear proteins [26,28]. The protease 2A^pro^ appears to be directly responsible for the degradation of the Nups as addition of the protease inhibitor, MPCMK abrogates the redistribution of nuclear resident proteins [80].

By contrast, cardiovirus-infected cells have hyperphosphorylated Nup62, Nup98, Nup153 and Nup214 (Figure 3C) [29,30]. Nup phosphorylation is L protein-dependent as expression of a zinc finger domain mutant L protein is unable to hyperphosphorylate Nups, whereas expression of the L protein alone does [81,82]. The L protein lacks any enzymatic function and evidence suggests that the L protein subverts host protein kinases to induce Nup phosphorylation by an unknown mechanism [81,82]. Another cardiovirus, Theiler’s murine encephalomyelitis virus (TMEV) prevents export of cellular mRNAs, which is indicated by the nuclear retention of polyA+ RNA by *in situ* hybridization in L protein expressing cells [30]. Interestingly, TMEV infection inhibits transcription of selected cytokine and chemokine genes, which are typically activated during viral infection [30]. In response to a viral infection, IRF-3 (IFN regulated factor-3) dimerizes and translocates to the nucleus to activate genes necessary to mount an anti-viral response. During a TMEV infection, the L protein is capable of blocking IRF-3 dimerization to antagonize host innate defenses [30]. It is interesting that enteroviruses degrade and cardioviruses hyperphosphorylate the same subset of Nups to alter the NPC function to acquire the necessary host nuclear proteins and to promote its cytoplasmic replication cycle.

## 5. Orthomyxoviruses

The *Orthomyxoviridae* family includes the influenza A virus, which causes annual epidemics of respiratory disease. The viral genome consists of eight single-stranded negative sense polarity RNAs, which are packaged with the nucleoprotein (NP) and the RNA-dependent RNA polymerase complex (PB2, PB1 and PA subunits) to form a viral ribonucleoprotein (vRNP) [83]. 

The influenza A virion is comprised of a lipid envelope studded with glycoprotein spikes of hemagglutinin (HA), which mediate virus attachment and entry by endocytosis [84]. In stark contrast to most other RNA viruses, replication and transcription of the influenza A virus genome occurs in the nucleus. Virion escape from the endosomal compartment releases the vRNP into the cytoplasm, where it is rapidly trafficked and imported into the nucleus (Figure 2C). The classical importin α/β transport system mediates import of the vRNP, although all the viral protein components of the vRNP have an NLS, it is unclear which protein is responsible for nuclear import. A number of studies have identified various members of the importin α family as interacting with NP and PB2 [31,32,33]. 

Independent genome-wide screens identify many host factors involved in nucleocytoplasmic transport that are crucial for influenza A virus, which include importin subunit β1 (KPNB1), CRM1, NXF1, Nup98 and Nup153 [34,35,40,47,85]. The precise roles of most of these hits in vRNP nuclear transport have yet to be defined. 

After replication, the RNA genome assembles into the vRNP nuclear export complex with the viral proteins: Nuclear Export Protein (NEP, also known as NS2) and the matrix protein M1 [86]. Indirect immunofluorescence of vRNP protein components indicates that LMB treatment causes retention of NP in the nucleus, suggesting that CRM1 plays a role in the export of the influenza A vRNP [36,37]. NEP contains an NES that is recognized by CRM1 and bridges the binding of CRM1 to the M1 protein [87,88,89]. However, a recent study using fluorescence *in situ* hybridization (FISH) analyses to localize virus mRNA and vRNA in influenza A-infected cells indicates that CRM1-depletion causes no significant difference in the export of influenza A virus RNA, whereas knockdown of NXF1 segregates influenza A virus mRNA and vRNA in the nucleus and adversely effects influenza A virus titers [38].

To date, influenza A virus appears to subvert two FG-Nups. Similar to HIV-1 [54], influenza A virus redistributes Nup62 to the cytoplasm in infected cells (Figure 3D), and Nup62 depletion sequesters viral mRNA and vRNA in the nucleus, which subsequently results in reduced virus titers [38]. Two-hybrid and luciferase assays indicate that the interferon-inducible protein Nup98 [90] interacts with NEP via its *N*-terminal FG-repeat domain [41]. Overexpression of the Nup98 GLFG‑repeat domain appears to act as a dominant negative, which results in decreased virus propagation [41], whereas knockdown of Nup98 has no significant effect on either virus replication or export of viral RNA from the nucleus [38]. Influenza A virus downregulates the expression of Nup98 (Figure 3D) and sequesters key constituents (NXF1, p15, Rae1, E1B-AP5) of the mRNA export machinery to selectively inhibit export of host mRNA that encode proteins related to host immune responses, such as IRF-1, MHC I and ICAM-1 [39,41]. Additionally, influenza A virus impairs the export of host CRM1 substrates from the nucleus as infected cells retain an NES-containing GFP fusion protein in the nucleus [86]. Taken together, influenza A virus appears to impair both host mRNA export machinery and CRM1-dependent export in order to favor virus replication and dampen the antiviral response.

## 6. Rhabdoviruses

Vesicular stomatitis virus (VSV) is the prototypical member of the *Rhabdoviridae* family and causes an acute vesicular disease in cattle, horses, swine and occasionally humans. This bullet-shaped, enveloped, single-stranded RNA virus encodes five proteins: the nucleoprotein (N), the phosphoprotein (P), the matrix protein (M), the glycoprotein (G) and the large polymerase protein (L) and replicates in the cytoplasm. VSV displays a broad tropism and robust infectivity that is mediated by its surface glycoprotein, VSV-G, binding to a somewhat disputed cell surface lipid (phosphatidylserine) or protein (LDL receptor) [91,92,93]. VSV attachment to the host cell results in internalization by endocytosis and escape of the vRNP into the cytoplasm upon endosomal pH acidification. The RNA-polymerase complex (N, P, L) produces the five viral transcripts in the cytoplasm and replicates the VSV genome. Finally, the virus assembles particles, which exit the cell by budding at the plasma membrane.

During a VSV infection, the M protein imposes a global blockade on host gene expression at the level of transcription, translation and mRNA export [94]. The M protein lacks any known enzymatic function and in addition to an important role in virion assembly and budding, it has been proposed to inhibit host gene expression by binding directly to host factors and altering their functions. In transfected cells, overexpression of a M-GFP fusion protein localizes to the nucleus and cytoplasm with a proportion of M-GFP colocalizing with NPC proteins at the NE [42,95]. 

The impact of M protein on nuclear import is a point of contention. While some evidence indicates that VSV blocks protein and RNP import [96,97], other data argue against this role [43,98,99]. Nonetheless, VSV exerts a clear effect on mRNA export with the M protein targeting the complex of Nup98 and the nuclear basket protein, Rae1 (Figure 3E) [42,43,100]. The M-Rae1-Nup98 complex interacts with chromatin in the nucleus [100]. VSV-infected cells depleted for Rae1 have unaltered levels of host mRNA nuclear accumulation, however in the absence of Rae1, VSV was unable to maintain its inhibition on host transcription [100]. Taken together, a new model proposes that the Rae1-Nup98 complex acts as a platform for M protein to interact with other host proteins essential for host transcription [100]. 

VSV subverts the NPC to evade a number of different host cell innate immune responses. Confocal microscopy analysis shows that the mRNA export factors, hnRNP A1, K and C1/C2 relocalize to the cytoplasm during VSV infection [99]. Interestingly, depletion of hnRNP A1 does not affect virus growth or protein synthesis, or the shutoff of host translation, however, VSV-infected cells progress to apoptosis more slowly than non-depleted cells [99]. In contrast to other viruses that promote replication by redistributing hnRNP A1 to the cytoplasm [101,102,103], the authors suggest that the Rae1‑dependent relocalization of hnRNP A1 is a host anti-viral response that induces hnRNP A1‑dependent expression of genes involved in upregulating apoptosis [99]. Many viruses block the IFN pathway as a means to evade the host’s innate immunity. IFN strongly inhibits VSV growth and therefore VSV has evolved to obstruct production of IFN in infected cells by specifically blocking the transport of a subset of host mRNAs. For example, transcription of IFN-β, IL-6 and c-Jun mRNA is induced by VSV infection and nearly 100% of these transcripts accumulate in the nucleus [100,104]. There is little evidence for the trapping of housekeeping gene mRNA, such as actin, in the nucleus due to the imposed VSV blockade of host transcription [100]. Inhibition of host gene expression at multiple levels, including nucleocytoplasmic transport by the VSV M protein provides the virus with a great advantage in evasion of host innate immune responses.

## 7. Retroviruses

Retroviruses are enveloped, positive-strand RNA viruses that synthesize complementary DNA (cDNA) by reverse transcription before incorporating this proviral cDNA into the host chromosome. Nuclear import of the proviral cDNA from gammaretroviruses, such as murine leukemia virus (MLV) occurs solely during cell division. By contrast, lentiviruses have a unique ability to access the nucleus in the absence of mitosis. This section focuses on the lentivirus human immunodeficiency virus 1 (HIV-1), the causative agent of acquired immunodeficiency syndrome (AIDS). 

The HIV-1 virion enters the host cell by membrane fusion, which is triggered upon binding of the envelope viral glycoprotein to the CD4^+^ receptor and a specific co-receptor (CXCR4 or CCR5) [105]. Uncoating of the incoming HIV-1 virion delivers two single-stranded RNA genomes into the cytoplasm for reverse transcription into cDNA, which associates with viral and cellular proteins to form the pre-integration complex (PIC) (Figure 2D) [106]. Several NLS-containing viral proteins have been implicated in nuclear translocation of the PIC such as integrase (IN), the viral protein R (Vpr) and the matrix (MA) protein, however their exact contributions have yet to be determined. IN interacts with soluble import receptors [107,108,109], while Vpr may play a role in targeting the PIC to the NPC [110,111]. However, accumulating experimental evidence suggests that the capsid (CA) protein is involved in driving PIC nuclear import [112,113]. 

A variety of large-scale screens identify a number of Nups and transport receptors that are modulated at the transcriptional and translational level in the presence of HIV-1. Quantitative proteomic screens of whole HIV-1 infected CD4^+^ and primary T cells pinpoint changes in the abundance of proteins involved in nucleocytoplasmic transport [114,115,116]. As a refinement on these studies, proteomic analyses on the NE of HIV-1-infected T cells demonstrate that the NPC undergoes extensive compositional changes in the presence of HIV-1 with a significant decrease in 18 of the 30 Nups without compromising the integrity of the NE or NPC [54]. High throughput RNA interference (RNAi) screens define a list of NPC components as being necessary host co-factor for HIV-1 infection, including Nup98, Nup85, Nup133, Nup107, Nup160, Nup153, Nup214, Nup358, Nup155, CRM1 (XPO1) and the nuclear import receptor transportin 3 (TNPO3) [46,47,49]. Additionally, a recent microarray study of mock and HIV-1-infected human primary CD4^+^ T lymphocytes indicates that Nup50 is downregulated and Nup62 is upregulated at 24 hours post-infection [52]. The functions of these nuclear transport factors and NPC proteins in HIV-1 infection have yet to be defined, although HIV-1 infectivity is clearly reduced upon depletion of Nup358 and TNPO3 [46,48,51,117,118,119]. Structural and biochemical analyses directly show that Nup358 interacts with CA to facilitate docking of the PIC at the NPC [44,118,120]. Additionally, the nuclear basket also appears to participate in PIC translocation by promoting exit into the nucleus [44]. Regardless of the contradictory evidence that surrounds Nup153 interacting with IN [45,121], CA seems to be the dominant determinate in Nup153 dependency as demonstrated by CA HIV-1 mutants being insensitive to Nup153 depletion and the ability of HIV-1 cores to interact directly with Nup153 [45,48,122,123].

It is interesting to note that the NPC is not only critical for nuclear import of the PIC but also plays a role in directing the integration of the proviral cDNA into the host chromosome (Figure 3F) [45,46,108,119,122,124]. Specifically, Nup62, Nup153 and Nup98 can be found in the nucleoplasm and have been shown to play a role in integration of viral cDNA into the host chromosome [44,53]. 

Splicing is required for nuclear export of mRNA but retroviruses produce unspliced, singly spliced and multiply spliced mRNAs and therefore need to export full-length transcripts that contain introns through the NPC [125]. The viral protein Rev mediates export of these intron-containing transcripts by binding to a specific sequence called the Rev response element (RRE). Rev encodes an NES, which serves to recruit CRM1 with Ran/GTP to the HIV-1 RNA RRE and together these form the viral ribonucleoprotein (vRNP) transport complex [50]. Evidence suggests that the vRNP interacts indirectly with Nup214, Nup153, Nup98 and Nup62 through different Rev co-factors to mediate nuclear export [126,127,128]. Indirect immunofluorescence demonstrates that lamin A/C is intact in HIV‑1-infected cells but a distinct redistribution of Nup62 from a NE localization to the cytoplasm (Figure 3F) [54]. Expression studies show that this dramatic relocalization is dependent on Rev‑mediated viral genomic RNA (vRNA) export [54]. Accompanying the cytoplasmic translocation is an apparent thickening of the Nup62 immunofluorescence signal at the NE [54]. Interestingly, immunogold staining of Nup62 and immunoblot analysis of purified virions identify Nup62 as an incorporated, virion-associated protein and suggests that Nup62 may be important for HIV-1 assembly or infectivity [54]. In agreement with this hypothesis, examination of vRNA localization by FISH in Nup62 depleted cells causes sequestration of vRNA in the nucleus, which is likely responsible for the observed decrease in viral gene expression, virus production and infectivity [54].

A number of host cell proteins are involved in HIV-1 nuclear transport at different stages in the virus life cycle, which appears to affect the localization of other host proteins. For example, HIV-1 imposes a blockade to the nuclear import of heterogeneous nuclear RNPs (hnRNPs) A1, A2 and D (AUF1) and the transport receptor, transportin 1 [103,129,130]. The RNA binding protein, hnRNP A1, shuttles between the nucleus and the cytoplasm and plays a role in pre-mRNA processing, metabolism and transport. Altering the composition of the NPC leads to the accumulation of hnRNP A1 in the cytoplasm and allows the virus to positively regulate HIV-1 internal ribosome entry site (IRES)‑mediated vRNA translation [103], similar to enterovirus 71 [101].

HIV-1 utilizes components of the NPC for both PIC import and Rev-vRNA RNP export but subversion of Nups may also a play key role in other cellular processes at various stages of HIV-1 replication, such as determining site of cDNA integration [45].

## 8. Hepadnaviruses

Hepadnaviruses are species-specific pathogens that are responsible for liver infections in humans and animals. Hepatitis B virus (HBV) is the best-known member of the *Hepadnaviridae* family. Studies with HBV are complicated by the lack of a convenient tissue culture model, forcing the use of duck HBV as a model for the human disease. 

A recent study suggests that HBV enters a transduced primary human hepatocyte cell line by clathrin-mediated endocytosis [131]. Upon escape of the virion from the endosomal system by membrane fusion, these cytoplasmic capsids range in size between 36 and 32 nm in diameter [132] and travel along microtubules toward the nucleus [133]. In the nucleus, the covalently closed circular genomic DNA is transcribed into pregenomic RNA (pgRNA). The pgRNA is exported into the cytoplasm via an unknown mechanism to act as a template for core protein and viral polymerase translation; it can also be encapsidated to form immature capsids. Mature infectious virions are enveloped and contain a partially double stranded DNA genome, which is generated by reverse transcription of the virion associated pgRNA [134]. Consequently, the cytoplasm can at once harbor both mature (DNA-containing) and immature (RNA-containing) virus particles.

As opposed to other DNA viruses, HBV capsids are imported intact through the NPC by an active mechanism and undergo uncoating once in the nucleus (Figure 2E) [3]. HBV particles are composed of NLS-containing capsid proteins, which only interact with importin α and β upon phosphorylation to expose the NLS sequences [135,136]. Mature and immature capsids are both transported into the nuclear basket of the NPC, however only mature capsids disintegrate to release the DNA genome into the nucleus while immature capsids are completely arrested in the basket (Figure 2E) [55,136,137]. The Nup153 FxFG-repeat domain binds HBV capsids and depletion of Nup153 causes a significant increase in the quantity of immature capsids within the nucleoplasm, suggesting that Nup153 is responsible for the selective release of the HBV genome [55]. Schmitz and colleagues examine protein translocation using NLS-BSA and M9-BSA constructs and demonstrate that immature arrested HBV capsids interfere with Nup153, which results in a blockade of the importin β-mediated nuclear import but not the transportin pathway [55].

## 9. Conclusions and Perspectives

Viruses, DNA viruses especially, need to import their genomes into the nucleus to propagate because they rely on the host’s DNA replication and/or transcription machineries. The NPC channel diameter imposes a size limitation for passive diffusion and demands active transport for viral genomes and their associated protein multiplexes. Virus families have evolved different strategies to breach the NPC but the restriction on size requires most viruses to shed their capsid before importing their genomes. The entirely or partially intact capsids of herpesvirus and adenovirus, respectively, dock at the NPC before uncoating to release their DNA genomes into the nucleus, whereas retrovirus and orthomyxovirus capsids undergo cytoplasmic uncoating steps that result in import of the viral genome (DNA or RNA) as a complex with viral proteins. By contrast, smaller hepadnavirus capsids remain intact during transit through the NPC, which allows uncoating and DNA release to occur once inside the nucleus. 

Different mechanisms of nuclear import are emerging as nuclear entry is studied more extensively. For example, the distantly related lentiviruses HIV-1 and FIV (feline immunodeficiency virus) appear to have distinct mechanisms of nuclear import [48]. This difference may stem from different specie‑specific viral tissue or co-factor requirements. It is interesting to note that a single mutation of one amino acid residue in HIV-1 CA can mimic the dependence of the FIV on certain Nups demonstrating a remarkable flexibility in HIV-1 nuclear transport as the virus can utilize various host factors for import [48]. This work particularly highlights the role of CA in nuclear transport, which has only recently been identified as the driving force behind PIC nuclear import [51]. Previously, IN, Vpr and MA had been implicated in nuclear transport as they all encode NLS signals and IN was shown to interact with various import receptors [138], implying that the presence of an NLS does not necessarily confirm a role in nuclear import and should be evaluated with caution.

Conflicting evidence also exists in terms of the host factors involved in transport. For example, there is a debate as to whether Nup214 or Nup358 is more important for HSV-1 docking to the NPC during infection [20,21]. The NPC is made up of multiple subunit complexes and the disruption of one can often affect the localization of another, which is true for the depletion of Nup214 causing a loss of Nup358 in the NPC [21,139]. The silencing of essential pore components can alter the overall structure of the NPC and complicate data interpretation.

Viral protein synthesis requires export of mRNA transcripts into the cytoplasm and newly synthesized viral proteins may require re-import in order to assemble virus particles in the nucleus, for example. To replicate efficiently, viruses require a large NPC trafficking capacity and may necessitate shutting down unnecessary host protein transport pathways, which also reduces the competition for host translational machineries in the cytoplasm. Imposing a blockade on the selective nuclear import is one way in which HIV-1 and poliovirus induce the mislocalization of certain nuclear proteins that they require for their replication.

Viruses alter the NPC composition using a variety of different mechanisms to facilitate nucleocytoplasmic transport. Many viruses target Nup153 and Nup62 for degradation or relocalization, which may be an important mechanism that drives the subversion of the NPC. Nup153 and Nup62 are required for the classical import pathway [140,141] but loss of these FG-Nups may disrupt other transport pathways as well. Recent work shows that Nups participate in a number of other cellular processes in addition to nucleocytoplasmic shuttling [142,143]. Subversion of Nups and inhibition of nucleocytoplasmic transport by viruses may be a key contributor to viral evasion of host antiviral response pathways. The interferon response pathway is known to require STAT (signal transducers and activators of transcription) nuclear import to activate genes encoding anti-viral functions. Therefore, preventing the import of STAT may be a strategy that viruses use to reduce the effectiveness of the interferon response [144]. Influenza A virus specifically blocks host mRNA export and downregulates the expression of the interferon inducible gene Nup98 [39]. Treatment with interferon induced the expression of Nup98 and restored mRNA transport, suggesting that modulating the expression of Nup98 might be a strategy to evade or antagonize innate immune responses [90]. Similarly, VSV specifically blocks the export of IFN-β, IL-6 and c-Jun mRNA, all of which are upregulated in VSV-infected cells [100,104]. Other virus proteins that affect a wide spectrum of host cell pathways including immune responses such as the NS5 (non-structural protein 5) of dengue virus might manipulate nuclear transport or NPC components.

In this review, we highlight the requirements for different host factors in the nuclear import and export of viral nucleic acids and proteins. The NPC is a key target of virus subversion and may provide the basis for developing antiviral compounds for therapeutic intervention that not only target viral proteins but also their interacting partners, karyopherins or Nups. 
